# British Columbia’s COVID-19 surveys on population experiences, action, and knowledge (SPEAK): methods and key findings from two large cross-sectional online surveys

**DOI:** 10.17269/s41997-022-00708-7

**Published:** 2022-12-02

**Authors:** Jat Sandhu, Ellen Demlow, Kate Claydon-Platt, Maritia Gully, Mei Chong, Megan Oakey, Rahul Chhokar, Gillian Frosst, Amina Moustaqim-Barrette, Sandy Shergill, Binay Adhikari, Crystal Li, Kari Harder, Louise Meilleur, Geoff McKee, Réka Gustafson, For British Columbia’s COVID-19 SPEAK working group

**Affiliations:** 1 British Columbia Centre for Disease Control, Vancouver, British Columbia Canada; 2 School of Population and Public Health, University of British Columbia, Vancouver, British Columbia Canada; 3 Vancouver Coastal Health, Vancouver, British Columbia Canada; 4 Vancouver Island Health Authority (Island Health), Victoria, British Columbia Canada; 5 Fraser Health, Surrey, British Columbia Canada; 6 Interior Health, Kelowna, British Columbia Canada; 7 Northern Health, Prince George, British Columbia Canada; 8 First Nations Health Authority, West Vancouver, British Columbia Canada

**Keywords:** COVID-19, Public health, Population health survey, Social determinants of health, Epidemiology, Methodology, COVID-19, santé publique, enquête sur la santé de la population, déterminants sociaux de la santé, épidémiologie, méthodologie

## Abstract

**Objectives:**

To describe the methodology and key findings of British Columbia’s (BC) COVID-19 SPEAK surveys, developed to understand the experiences, knowledge, and impact of the COVID-19 pandemic on British Columbians.

**Methods:**

Two province-wide, cross-sectional, web-based population health surveys were conducted one year apart (May 2020 and April/May 2021). Questions were drawn from validated sources grounded within the social determinants of health to assess COVID-19 testing and prevention; mental and physical health; risk and protective factors; and healthcare, social, and economic impacts during the pandemic. Quota-based non-probability sampling by geography was applied to recruit a representative sample aged 18 years and older. Recruitment included strategic outreach and longitudinal follow-up of a subgroup of respondents from round one to round two. Post-collection weighting using Census data by age, sex, education, ethnicity, and geography was conducted.

**Results:**

Participants included 394,382 and 188,561 British Columbians for the first and second surveys, respectively, including a longitudinal subgroup of 141,728. Key findings showed that societal impacts, both early in the pandemic and one year later, were inequitably distributed. Families with children, young adults, and people from lower socioeconomic backgrounds have been most impacted. Significant negative impacts on mental health and stress and a deterioration in protective resiliency factors were found.

**Conclusion:**

These population health surveys consisting of two large cross-sectional samples provided valuable insight into the impacts and experiences of British Columbians early in the pandemic and one year later. Timely, actionable data informed several high-priority public health areas during BC’s response to the COVID-19 pandemic.

## Résumé

**Objectifs:**

Décrire la méthode et les principaux constats des enquêtes SPEAK de la Colombie-Britannique sur la COVID-19, élaborées pour comprendre l’expérience des Britanno-Colombiens durant la pandémie, ainsi que leurs connaissances de la pandémie et les effets qu’elle a eus sur eux.

**Méthode:**

Deux enquêtes en ligne transversales sur la santé de la population ont été menées dans toute la province à un an d’intervalle (en mai 2020 et en avril-mai 2021). Les questions, qui provenaient de sources validées ancrées dans les déterminants sociaux de la santé, ont servi à évaluer le dépistage et la prévention de la COVID-19; la santé mentale et physique; les facteurs de risque et de protection; et les effets sociaux, économiques et sur les soins de santé ressentis durant la pandémie. Un échantillonnage contingentaire non probabiliste par lieu géographique a été appliqué pour recruter un échantillon représentatif de personnes de 18 ans et plus. Le recrutement a inclus une prise de contact stratégique et un suivi longitudinal auprès d’un sous-groupe de répondants entre les cycles un et deux. Après la collecte, les données ont été pondérées selon l’âge, le sexe, le niveau d’instruction, l’ethnicité et le lieu géographique à l’aide des données du Recensement.

**Résultats:**

Les participants étaient 394 382 Britanno-Colombiens au cours du premier cycle de l’enquête et 188 561 au deuxième cycle, dont un sous-groupe longitudinal de 141 728 personnes. Selon les principaux constats, la répartition des effets sociétaux, tant au début de la pandémie qu’un an plus tard, a été inéquitable. Les familles avec enfants, les jeunes adultes et les personnes de statut socioéconomique plus faible ont été les plus touchés. D’importants effets nuisibles sur la santé mentale et le stress ont été constatés, ainsi qu’une détérioration des facteurs de résilience protecteurs.

**Conclusion:**

Ces enquêtes sur la santé de la population comprenant deux grands échantillons transversaux ont jeté un éclairage précieux sur les effets subis et les expériences vécues par les Britanno-Colombiens au début de la pandémie et un an plus tard. Ces données opportunes et exploitables ont éclairé plusieurs domaines hautement prioritaires de la santé publique durant la riposte de la Colombie-Britannique à la pandémie de COVID-19.

## Introduction

The 2019 SARS-CoV-2 (COVID-19) pandemic has caused unprecedented disruption and challenges worldwide. In response, broad public health surveillance and response measures have been implemented to minimize transmission and protect individuals susceptible to severe disease while limiting societal disruption. Despite highly effective vaccines, COVID-19 continues to spread globally, resulting in the prolonged implementation of stringent public health measures.

The broad impacts of the COVID-19 pandemic and the public health response measures have greatly affected everyday life, including physical, mental, social, and economic well-being (Douglas et al., 2020; Wang et al., 2020; World Health Organization, 2020). These impacts have further exacerbated disparities in health outcomes and determinants of health and vulnerabilities within our healthcare system. Many segments of the population that already experience inequities, including people with low socioeconomic status, visible minorities and marginalized groups, young adults, and families with children, have been disproportionally affected (Munasinghe et al., 2020; Samji et al., 2021; Wang et al., 2020).

The wide-reaching societal impacts and interventions related to the pandemic have driven the need for dynamic population health surveillance to understand and address the societal consequences of the pandemic. Large-scale representative population health surveys can provide reliable insight into individuals’ experiences and inform the public health response and societal changes needed to support the health and well-being of the population and reduce health inequities (World Health Organization Regional Office for Europe, 2020). Due to the significant variation in the transmission of SARS-CoV-2 and public health response strategies across Canadian and international jurisdictions, there was a need for a comprehensive and representative survey to inform public health services and pandemic response measures within British Columbia (BC), Canada.

The BC COVID-19 Survey on Population Experiences, Action and Knowledge (SPEAK) measured the populations’ perceptions of risk, acceptability of the public health response and recovery measures, and the broader impacts of the COVID-19 pandemic at local, regional, and provincial levels. The initial survey (round one) assessed BC residents’ experiences during the early stages of the pandemic to inform ongoing public health measures and assess the unintended consequences. The second survey (round two) was conducted a year later to assess the changes in behaviours and experiences since the early phase of the pandemic; understand barriers to vaccination; inform health and social and economic investment during the pandemic recovery; and assess inequities across different population groups.

This paper provides an overview of the methods used to develop the population health surveys, key findings, and how these findings have informed public health initiatives during the COVID-19 pandemic in BC.

## Methods

### Study design and data collection

An observational cross-sectional study design assessed the experiences of the COVID-19 pandemic among the adult population in BC, Canada, at two specific time points: 12 May–31 May 2020 and 8 April–9 May 2021.

### Survey development

The two SPEAK surveys were designed and implemented using the same methodology to answer questions relevant to specific pandemic stages, sharing many core questions and enabling cross-sectional comparisons over time. A working group was formed with public health leaders across BC, including provincial and regional organizations representatives, to share knowledge and local perspectives.

Targeted literature reviews and environmental scans provided the theoretical basis for the domains of interest, survey objectives, and questions. Key survey domains reflected the survey’s overarching goals, and multiple questions were selected or developed to represent each domain. The Social Determinants of Health Model (Whitehead & Dahlgren, 2006) informed the development and selection of the domains and questions. This model is relevant to understanding health inequities, public health priority areas, and the unintended impacts of the pandemic and public health response measures.

The initial survey covered eight domains: socio-demographics; COVID-19 response, testing, and prevention; experience; risk and protective factors; healthcare; social; economic; and resiliency. The second survey encompassed ten domains: the eight from round one and the added domains of vaccine and adaption. Survey questions were primarily selected or adapted from publicly available or validated tools:

- Statistics Canada (Statistics Canada, 2009, 2018, 2020a, 2020b, 2020c, 2020d, 2020e; Statistics Canada, Mexico’s Instituto Nacional de Estadística y Geografía (INEGI), & Economic Classification Policy Committee (ECPC) of the United States Office of Management and Budget, 2017);
- Canadian and International Health Surveys (Carman et al., 2020; Johns Hopkins University, 2020; Ogilvie et al., 2021; United Kingdom Office for National Statistics, 2021; University of California Los Angeles (UCLA), 2004, 2020a, 2020b; Vancouver Coastal Health, Fraser Health, & University of British Columbia, 2020); and
- World Health Organization (World Health Organization Regional Office for Europe, 2020).

Additionally, the working group created several novel questions. Questions were reviewed and selected relevant to public health priority areas. The round one survey consisted of 85 questions; 52 of the 85 were retained for round two, and a further 50 questions were added, resulting in 102 questions (Table 1). The questions added in round two evaluated attitudes related to vaccination, pandemic adaption, and other mental health and societal impacts. All questions were categorical, except for two open-ended questions to further explore respondents’ experiences. Aside from age, sex, and geographic indicators, all questions were optional and included a “prefer not to answer” response option.

**Table 1:** Composition of the BC COVID-19 SPEAK surveys

Survey round one: May 2020 (85 questions)		Survey round two: April/May 2021 (102 questions)	
Domain	Content	Domain	Content
Socio-demographics	Age, gender, education, household income, ethnicity, household composition	Socio-demographics	Age, sex, gender, education, household income, ethnicity, household composition
Response and prevention	Preventive personal behaviours, self-isolation, sick leave, work remotely, physical distance at work, public health response appropriate	COVID-19 testing, response, and prevention	Tested for COVID-19, never been tested, avoided testing, contact by public health, self-isolation, work remotely, follow public health, visit non-essential business
Experience	Feel helpless, concern due to pandemic, mental health, stress, feel control	Experience	Mental health, stress, concern due to pandemic, feeling lonely, hopeful for future, impact on mental healthcare
Risk and protective factors	Walk/run/cycle for recreation and commute, public transit, fruit and vegetable consumption, sleep, alcohol intake, cannabis use, smoking, one or more health conditions associated with risk for COVID-19	Risk and protective factors	General health, walk/run/cycle for recreation and commute, public transit, physical activity, fruit and vegetable consumption, sleep, alcohol intake, binge drinking, cannabis use, smoking, one or more health conditions associated with risk for COVID-19
Healthcare impacts	Difficulty accessing or avoiding healthcare, virtual care interest	Healthcare impacts	Difficulty accessing or avoiding healthcare, impact on health
Social impacts	Childcare, children impaired learning, stress, screen time, connection with friends	Social impacts	Impact on employment, education, and housing, discrimination and setting, household conflict, childcare, child well-being, physical activity, stress, screen time, fruit and vegetable consumption, connection with family and friends, learning, sleeping, and extracurricular activities, school open, school notification, impact on post-secondary education
Economic impacts	Visiting non-essential business, not working, work impaired, financial difficulty — current and future, food insecurity, house insecurity	Economic impacts	Not working, studying, work interruption, work impaired, financial difficulty — current and future, food insecurity, house insecurity, financial relief services, living arrangement
Resiliency	Connection with family and friends, community belonging	Resiliency	Connection with family and friends, community belonging
		Vaccine	Vaccine eligibility, vaccine hesitancy, received vaccine, beliefs of vaccines
		Adaption	Concern resuming activities, areas to see change, areas to continue

### Survey delivery and testing

Qualtrics (Qualtrics, 2020), an online survey tool, was used to deliver both surveys. A web-based survey was chosen over paper or telephone survey methods to facilitate cost-effective and rapid development, data collection, and analysis and reduce manual entry error. A call centre was also established for round one to assist individuals who needed support to complete the survey; this assistance was not offered in round two due to low uptake. The surveys were available in English and Simplified Chinese for round one, and French and Punjabi were added in round two. Language guides were available for both survey rounds in French, Punjabi, American Sign Language, Korean, Spanish, Vietnamese, Farsi, Arabic, and Chinese.

Pre-testing of the survey was conducted with members of the working group to evaluate face validity, comprehension, content, layout, and design. Technical aspects were tested to maximize accessibility and compatibility across most platforms (smartphones, computers, and tablets) and internet browsers. Completion times in English, French, Chinese, and Punjabi were assessed, averaging 10–20 min (round one) and 20–30 min (round two).

### Participants, sampling, and recruitment

Both surveys’ target population encompassed all residents of BC aged 18 years and older. A non-probability quota-based sampling method was used, rather than probability random sampling methods, as it was the most time-efficient and inexpensive way to obtain the information required (Groves et al., 2009). To ensure that representative samples were obtained across different geographic regions of BC, sampling quotas were calculated for age, sex, income, education, and ethnicity (Appendix 1 and Appendix 2). The areas were defined by BC health administrative boundaries, using hierarchical categorization of data ordered from most to least granular: Community Health Service Area (CHSA), Local Health Area (LHA), Health Service Delivery Area (HSDA), Health Authority (HA), and the Provincial (BC) level.

Using Census data, sample size calculations were performed for each CHSA by age and sex (Statistics Canada, 2016). HSDA targets were determined by either the crude target (2% of the urban population or 4% of the rural population determined by CHSA population density rank) or the sample size based on the hypergeometric distribution with a 4% margin of error, whichever was larger (Appendix 1 and Appendix 2). Progress toward recruitment targets was monitored daily; however, outreach was limited due to pandemic response measures. In addition, 250,901 respondents from the initial survey who provided an email for follow-up were invited to participate in round two.

### Statistical methods

Statistical analysis was performed using Statistical Analysis System (SAS version 9.4) (SAS Institute Inc, 2008) and R (version 3.6.2) (R Core Team, 2013) statistical software packages.

#### Data preparation

The data were cleaned to improve overall data quality. Duplicate surveys and those with missing age, sex, and geography data were removed. A minimum degree of progression through the survey was required for inclusion in the final analytical dataset. Cut-off points were determined by assessing the natural attrition points of survey progression. After review, a cut-off point for survey progression was selected at 31% for round one and 33% for round two. Data were also suppressed for geographical areas with more than 25% Indigenous population in accordance with Indigenous data governance practices.

#### Weighting

Post-collection statistical weighting was performed to minimize potential biases introduced by the study design and sampling methods and to ensure the results were representative of the BC population using Census data by geography (HSDA, LHA, and CHSA) based on age, sex, education, and ethnicity questions (Appendix 3). Stratifications were limited while optimizing representativeness across the survey region. The weighted values were calculated as percentages with corresponding 95% confidence intervals (CI). Coefficients of variation were calculated and estimates greater than 33.3% were considered unreliable and were suppressed.

#### Validation of sample

Several questions were derived from the Canadian Community Health Survey (CCHS) (Statistics Canada, 2018, 2020a). The CCHS is a large cross-sectional survey using a rigorous methodology and a probability random sampling method to provide representative health region–level estimates every 2 years. However, CCHS may be subject to selection bias, as it is conducted by phone in English or French. Comparisons between pre-pandemic indicators (non-communicable conditions, mental health, social connectedness, and lifestyle risk factors) from the 2017/2018 CCHS (Statistics Canada, 2018) and the SPEAK surveys were conducted to contextualize and assess the representativeness of the SPEAK survey samples during the pandemic.

## Results

### Sample population

In total, 394,382 (round one) and 188,561 (round two) individuals were included in the analytical datasets, providing a large and comprehensive sample of the BC population (approximately one in ten and one in twenty-five people aged 18 years and over residing in the province, respectively) (Table 2). A total of 250,901 round one participants who provided their contact details were invited to participate in round two; 148,452 (59.2%) responded. A total of 141,728 survey responses were included in the final dataset, providing longitudinal data to assess changes between the survey rounds at individual and population levels. The sample was weighted using the 2016 census data, and the unweighted and weighted samples of rounds one and two of the BC COVID-19 SPEAK surveys are shown in Table 3.

**Table 2:** Overall response rate by survey round

Survey round	No. of responses		Excluded surveys***	Total surveys in final dataset
	Invited to participate R2*	Self-enrolled through website		
Round one	N/A	433,754	39,372 (9.1%)	394,382
Round two	148,452	57,789	17,680 (8.6%)	188,561

*Participants who self-enrolled in round one survey and participated in round two surveys through email invitation

**Excluded data missing on gender (round one) or sex (round two), age and geography or did not meet the survey progression cut-off of 31% (round one) or 33% (round two)

**Table 3:** Unweighted and weighted samples of rounds one and two of the BC COVID-19 SPEAK survey

Demographics	BC COVID-19 SPEAK survey				2016 Census (%) population (BC)
	Round one (2020)		Round two (2021)		
	Unweighted sample (%)	Analytic sample Weighed (%)	Unweighted sample (%)	Analytic sample Weighed (%)	
Sex					
Female	70.2	51.9	70.8	51.4	51.5
Male	29.8	48.1	29.2	48.6	48.5
Age (years)					
18–34	19.8	28.2	14.2	27.9	26.9
35–54	37.7	34.2	35.3	34.4	33.7
55–74	36.3	30.0	43.0	30.0	31.0
≥ 75	6.2	7.7	7.5	7.7	8.4
Visible minority*					
Not a visible minority	80.8	66.1	85.9	68.7	65.8
Chinese	4.4	10.7	4.1	10.6	11.2
South Asian	2.4	6.6	2.1	6.8	7.5
Indigenous	2.7	3.4	3.2	4.6	5.0
Others	8.8	10.7	4.7	9.3	10.5
Education					
Below high school	2.0	11.1	1.4	10.8	12.5
High school	15.2	30.6	12.5	30.6	30.2
Certificate or diploma	34.2	32.4	33.8	32.3	31.6
University degree	48.7	25.9	52.2	26.2	25.6

In both survey rounds, the response rates were surpassed for the crude provincial target based on sample size calculations and population targets for each of the five HAs (Appendix 1 and Appendix 2). Response rates far exceeded aggregate sample size calculations at a provincial level. However, rural communities, populations with lower educational attainment, lower household incomes, and visible minorities did not meet the HA sample size calculations.

Comparisons of several indicators between the SPEAK and the CCHS samples are shown in Table 4. Self-reported comorbidities of the SPEAK samples for diabetes, heart disease, and cancer (types not specified for each condition) were similar to the 2017/2018 CCHS sample. Self-perceived general health as poor or fair was similar in magnitude across the three BC samples (CCHS and both survey rounds), although slightly higher in the CCHS sample.

**Table 4:** Comparison of the BC COVID-19 SPEAK survey samples and the Canadian Community Health Survey by key indicators

Demographics	BC COVID-19 SPEAK survey		2017/18 Canadian Community Health Survey (%) BC population
	Round one (2020)	Round two (2021)	
	Analytic sample Weighed (%)	Analytic sample Weighed (%)	
Diabetes*	7.3 (7.0, 7.5)	7.0 (6.7, 7.4)	6.2 (5.7, 6.7)
Heart disease*	5.7 (5.4, 5.9)	5.2 (4.9, 5.5)	4.8 (4.3, 5.2)
Cancer*	6.1 (6.0, 6.3)	6.4 (6.2, 6.7)	6.7 (5.7, 6.7)
General health — fair/poor	9.8 (9.5, 10.1)	10.6 (10.2, 11.0)	12.9 (12.1, 13.8)
Mental health — fair/poor	19.5 (19.2, 19.8)	32.1 (31.6, 32.7)	8.9 (8.1, 9.7)
Increased stress — quite/extremely	18.3 (18.0, 18.6)	24.9 (24.4, 25.5)	21.6 (20.5, 22.7)
Weak sense of community belonging	35.5 (35.1, 35.8)	53.9 (53.3, 54.5)	30.1 (28.8, 31.3)
Moderate physical activity — 150+ mins/week	N/A	69.1 (68.5, 69.6)	64.8 (63.5, 66.0)
Smoking daily or occasionally**	14.8 (14.6, 15.1)	13.7 (13.3, 14.2)	13.3 (12.5, 14.2)**

*Self-reported and type not specified

**Does not specifically include vaping

Self-perceived mental health as poor or fair was notably higher at both time points (start of the pandemic and one year later) than the proportion of the BC population who reported poor or fair mental health during 2017/2018. Similar findings for community belonging showed a small weakening at the start of the pandemic compared to the CCHS sample, and this proportion decreased further a year later. The deterioration in mental health and social connectedness are consistent with the current literature relating to the negative impacts arising from the pandemic.

Moderate physical activity of 150 min or greater per week and smoking daily or occasionally were comparable to the CCHS sample.

### Key findings

The round one survey showed that, during the early stages of the COVID-19 pandemic, the negative societal impacts were not distributed equitably; the greatest impact was experienced by those with the fewest resources and already experiencing the greatest stress. One year into the pandemic, the second round of the survey showed a further deterioration in health, social and economic impacts, and resiliency, disproportionately affecting those with poorer social determinants of health.

#### Mental health

There was an increase in the proportion of British Columbians who self-perceived their mental health as poor or fair between survey rounds, and there was a further indication of a decline in mental health with an increase in people who reported worsening mental health (Table 5). There was also an increase in perceived life stress as quite stressful or extremely stressful. Communities across BC reported experiencing a significant increase in reduced connections to family and friends. There was also an increase in people reporting a weak sense of community belonging.

**Table 5:** BC COVID-19 SPEAK survey — mental health impacts by survey round

Indicator	BC COVID-19 SPEAK survey	
	Round one	Round two
	BC overall (%)	BC overall (%)
Mental health — poor/fair	19.5 (19.2, 19.8)	32.1 (31.6, 32.7)
Mental health worsening	46.4 (46.1, 46.8)	57.1 (56.5, 57.7)
Increased stress — quite/extremely	18.3 (18.0, 18.6)	24.9 (24.4, 25.5)
Difficulty accessing mental healthcare	N/A	14.4 (13.3, 15.5)
Decreased connection with family	42.4% (42.1, 42.7)	57.1 (56.5, 57.6)
Decreased connection with friends	61.4 (61.0, 61.7)	77.3 (76.7, 77.8)
Weak sense of community belonging	35.5 (35.1, 35.8)	53.9 (53.3, 54.5)

Note. The indicator ‘Difficulty accessing mental healthcare’ was not asked in round one

#### Young adults

There were significant impacts on young adults aged 18–29 years throughout the pandemic, with substantial disruptions to their mental health, employment, financial security, and life goals (Table 6). Compared to all adults, people aged 18–29 years reported a greater impact on their mental health, with a greater deterioration since the pandemic’s start. They were also twice as likely to report increased difficulty accessing mental healthcare than all adults. A weak sense of connection to their community also increased during the surveys. Current and future financial stress remained high, despite almost three quarters of people reporting they had accessed financial supports or services. Housing and food insecurity remained high between the two surveys for this age group.

**Table 6:** BC COVID-19 SPEAK survey — young adults (18–29 years) and all adults (18+ years)

Indicator	BC COVID-19 SPEAK survey			
	Round one		Round two	
	Young adults (%)	BC overall (%)	Young adults (%)	BC overall (%)
Mental health worsening	54.2 (53.3, 55.1)	46.4 (46.1, 46.8)	66.3 (64.7, 68.0)	57.1 (56.5, 57.7)
Increased stress — quite/extremely	23.2 (22.4, 24.0)	18.3 (18.0, 18.6)	38.3 (36.6, 39.9)	24.9 (24.4, 25.5)
Difficulty accessing mental healthcare	N/A	N/A	31.0 (28.3, 33.6)	14.4 (13.3, 15.5)
Weak sense of community belonging	49.8 (48.8, 50.8)	35.5 (35.1, 35.8)	68.6 (66.9, 70.3)	53.9 (53.3,54.5)
Not working due to the pandemic	26.9 (26.1, 27.8)	15.5 (15.3, 15.8)	7.9 (6.9, 8.9)	5.1 (4.8, 5.3)
House insecure	8.3 (7.7, 8.8)	5.2 (5.0. 5.4)	9.4 (8.4, 10.4)	5.3 (4.9, 5.6)
Food insecure	19.1 (18.3, 19.9)	15.6 (15.3, 15.9)	20.2 (18.6, 21.9)	12.3 (11.8, 12.9)
Current financial stress	41.7 (40.8, 42.6)	32.3 (32.0, 32.7)	43.0 (41.2, 44.8)	28.8 (28.3, 29.4)
Future financial stress	52.9 (51.9, 53.8)	42.6 (42.3, 43.0)	39.0 (37.2, 40.8)	26.1 (25.5, 26.7)
Use of financial supports	N/A	N/A	71.5 (69.9, 73.0)	46.3 (45.7, 46.9)

Note. The indicators ‘Difficulty accessing mental healthcare’ and ‘Use of financial supports’ were not asked in round one

#### Households with children

Since the beginning of the pandemic, a greater proportion of households with children reported worsening mental health and financial security than households without children and the negative impacts on their children’s stress and social connections (Table 7). Both types of household compositions reported a weak connection to their community. Parents reported increased stress for children aged 5–17 years throughout the pandemic and reduced social interaction with friends.

**Table 7:** BC COVID-19 SPEAK survey — households with children

Indicator	BC COVID-19 SPEAK survey			
	Round one		Round two	
	Households with children (%)	Households without children (%)	Households with children (%)	Households without children (%)
Mental health worsening	51.0 (50.4, 51.7)	44.5 (44.1, 44.9)	62.4 (61.2, 63.6)	55.2 (54.5, 55.9)
Increased stress — quite/extremely	23.9 (23.3, 24.4)	15.6 (15.3, 15.9)	30.2 (29.1, 31.2)	22.7 (22.0, 23.4)
Weak sense of community belonging	34.1 (33.4, 34.9)	36.0 (35.5, 36.4)	53.2 (52.0, 54.4)	54.0 (53.3, 54.6)
Not working due to the pandemic	17.1 (16.6, 17.6)	14.8 (14.4, 15.1)	4.6 (4.2, 5.1)	5.2 (4.9, 5.5)
House insecure	5.6 (5.2, 5.9)	4.9 (4.7, 5.1)	5.3 (4.3, 5.5)	4.9 (4.8, 5.8)
Food insecure	17.9 (17.3, 18.4)	14.3 (14.0, 14.6)	14.6 (13.4, 15.8)	11.3 (10.8, 11.9)
Current financial stress	39.1 (38.4, 39.7)	29.1 (28.7, 29.5)	34.4 (33.2, 35.6)	26.5 (25.9, 27.1)
Future financial stress	49.5 (48.8, 50.1)	39.4 (39.0, 39.8)	30.3 (29.1, 31.5)	24.4 (23.7, 25.0)

#### Healthcare access

British Columbians reported increased difficulty accessing healthcare since the start of the pandemic, and of those who reported difficulty accessing healthcare, the family doctor, dentist, and diagnostic services were most frequently reported as difficult to access (Table 8). Respondents also reported an increase in avoiding healthcare, with family doctors cited as the most avoided type of healthcare service. In round two, 39.7% of respondents reported their health worsened due to difficulty accessing or avoiding healthcare.

**Table 8:** BC COVID-19 SPEAK survey — healthcare impacts

Indicator	BC COVID-19 SPEAK survey	
	Round one	Round two
	BC overall (%)	BC overall (%)
Difficulty accessing healthcare	22.6 (22.3, 22.9)	36.2 (35.6, 36.8)
Difficulty — family doctor	51.8 (51.1, 52.6)	77.9 (77.2, 78.7)
Difficulty — dentist	55.5 (54.5, 57.4)	33.2 (32.1, 34.3)
Difficulty — diagnostic services	22.1 (21.4, 22.8)	28.2 (27.1, 29.4)
Avoiding healthcare	33.3 (33.0, 33.6)	41.6 (41.0, 42.2)
Avoiding — family doctor	61.9 (61.4, 62.4)	64.6 (63.7, 65.5)
Avoiding — dentist	55.5 (54.8, 56.3)	53.6 (52.6, 54.5)
Avoiding — diagnostic services	20.2 (19.6, 20.7)	19.7 (18.9, 20.5)

#### Vaccine uptake and beliefs

A total of 9.2% reported vaccine hesitancy, with higher levels of vaccine hesitancy reported across some health regions, with Northern HA reporting twice the amount of hesitancy than overall BC, households with an income of less than $20K, individuals with below high school and high school level of education, and people from West Asian or Arab ethnic backgrounds reported higher levels of vaccine hesitancy (Table 9). There was high agreement among respondents in the belief that COVID-19 vaccines were beneficial (89.8%), safe (80.1%), and helpful to get back to everyday life (85.7%).

**Table 9:** BC COVID-19 SPEAK survey — vaccine hesitancy

Indicator		BC COVID-19 SPEAK survey
		Round two % (CI)
		Vaccine hesitant*
Health region	Interior	11.7 (10.7, 12.7)
	Fraser	9.7 (9.1, 10.4)
	Vancouver Coastal	6.3 (5.8, 6.9)
	Vancouver Island	7.1 (6.6, 7.7)
	Northern	18.1 (15.5, 20.7)
Education	Below high school	10.4 (8.1, 12.6)
	High school	10.6 (9.8, 11.4)
	Certificate or diploma	10.1 (9.7, 10.5)
	University degree	5.7 (5.4, 5.9)
Income	<$20K	11.7 (8.8, 14.6)
	$20K–$59K	9.7 (8.9, 10.6)
	$60K–$99K	9.7 (8.7, 10.7)
	$100K–$139K	9.1 (8.4, 9.9)
	$140K+	7.6 (7.0, 8.2)
Ethnicity	White	8.9 (8.5, 9.2)
	Chinese	6.9 (5.7, 8.1)
	South Asian	8.3 (5.9, 10.8)
	Southeast Asian/Filipino	8.3 (5.6, 10.9)
	West Asian/Arab	13.2 (9.2, 17.3)
	Japanese/Korean	7.6 (4.4, 10.8)
	Black	10.0 (5.6, 14.3)
	Latin-American/Hispanic	10.9 (8.2, 13.6)
	Multiple/other	13.0 (11.2, 14.8)

*9.2% of the adult BC population

#### Adaption

Most British Columbians reported they would like more flexible work options to continue post-pandemic (75.0%); this was highest in people aged 18–50 years (18–29: 80.3%, 30–39: 81.8%, 40–49: 79.2%). The majority (65.2%) of British Columbians also reported wanting to maintain increased access to virtual care; this was highest in people aged 30–69 years (30–39: 69.3%, 40–49: 69.5%, 50–59: 68.8%, 60–69: 65.6%). Most BC respondents would also like to see societal changes to include greater healthcare access (70.9%), reduced income inequality (56.0%), and expansion of green space (54.4%).

#### Uses of BC COVID-19 SPEAK data

Key indicators were available several months after the closure of the surveys on publicly available dashboards (British Columbia Centre for Disease Control, 2020). Public health decision-makers used key findings to inform policy and prioritize support and public health initiatives to:

- Inform re-opening plans for safe return to school for kindergarten to grade 12 (Dove et al., 2020) and the return of in-person post-secondary education.
- Model vaccine projections, inform and target interventions to areas with high rates of vaccine hesitancy, and inform COVID-19 vaccine program decisions and equity considerations.
- Raise discussions with medical and health leaders around virtual health and healthcare access appropriateness.
- Raise discussions with community stakeholders to target support and initiatives to improve mental health.
- Inform recovery priorities in supporting the health and well-being of young adults aged 18–29 years (Samji et al., 2021).

## Discussion

The two BC COVID-19 Population Health Surveys represent some of the most extensive known assessments of the societal impacts of the COVID-19 pandemic in Canada and internationally. A consistent and rapid development process at each time point enabled the collection of time-sensitive, population-representative data on many important public health indicators during an evolving pandemic. The short timeline from conception, through development, validation, deployment, analysis, and dissemination, enabled population-specific contemporaneous data to be available to public health decision-makers to inform public health policy, active response, and recovery planning.

The key findings demonstrated both breadth of societal impact and significant inequity in the distribution of negative societal impacts resulting from the pandemic and the public health response early in the pandemic and one year later. The pandemic has disproportionately affected people already experiencing the greatest stresses, notably young adults, families with children, people in lower-income groups, and some ethnic minority groups. The extensive negative impact on physical and mental health, social connectedness, and economic stability as well as resiliency were consistent with the findings of other studies (Douglas et al., 2020; Munasinghe et al., 2020; Statistics Canada, 2018, 2020a; Wang et al., 2020; World Health Organization, 2020). These findings provided insight into the magnitude and distribution of the impact among British Columbians during the pandemic and helped inform several high-priority public health decisions in BC.

### Strengths and limitations

There are inherent strengths and limitations to using a cross-sectional study design and a non-randomized quota-based sampling method for rapid population health assessment. Sample sizes were very large for both rounds of the survey; however, some limitations may affect the generalizability of the results and should be considered when interpreting the findings.

A cross-sectional observational study design was used to provide descriptive population data that allowed different population groups and characteristics to be compared at a single point in time. This study design was chosen over other designs, such as a longitudinal study, to provide an informative snapshot of the public disposition quickly and inexpensively in a dynamic and evolving environment; however, due to the inherent limitations of cross-sectional study designs, causal associations cannot be drawn.

A non-probability quota-based sampling method was used to target recruitment for each geographic area for age, sex, income, education, and ethnicity rather than probability random sampling methods. This sampling method was time-efficient and inexpensive to obtain relevant data and meet sample size targets, and the sample size targets for each geographic area were exceeded in both surveys. Despite active and targeted recruitment, some demographic groups and population segments may have been under-represented in the survey. In addition, the option to distribute the survey electronically using a web-based tool may have led to the under-representation of some groups or population segments based on limited internet connectivity, technological proficiency, or geographical location.

Post-collection weighting with Canadian Census estimates by geographic level for age, sex, education, and ethnicity was used to account for the residual differences within the samples and help to minimize bias. The survey data are matched to the 2016 Census estimates. Although the Census may have changed over the last 4 years, the 2016 Census was the best source and most recent to create a population-representative sample. One outcome of the sampling method was that the prevalence of comorbidities (diabetes, heart disease, and cancer) was consistent with those of randomized samples for the BC population reported by CCHS. When comparing several indicators in the SPEAK sample with the CCHS, differences were seen pre-pandemic and throughout the pandemic, which are reflective and consistent with the literature indicating good representativeness of the weighted samples.

### Future work

The analysis of the longitudinal subpopulation data to understand the change between the two time points at an individual and population level and qualitative analysis of the open-ended questions will provide an invaluable perspective. Further health assessment of the BC population is needed to understand and address the health, social, and economic impacts and inequities among health outcomes across different population groups. This work will be critical for pandemic recovery.

## Conclusion

These population health surveys conducted twice during the COVID-19 pandemic across BC are the most extensive and representative population studies to date. The surveys delivered timely, actionable data to help decision-makers address the burden of the COVID-19 pandemic in BC, informing several critical public health priority activities. As the direct and indirect consequences of the COVID-19 pandemic continue, monitoring and understanding these impacts will be essential over time. This survey methodology provides a rapid and responsive process for population health assessments to inform public health interventions, practices, and policies.

## Contributions to knowledge

What does this study add to existing knowledge?

- The extensive population health surveys consisting of cross-sectional samples of the BC adult population provided insight into the experiences and societal consequences of the COVID-19 response early in the pandemic and one year later.
- The surveys captured the effect of the pandemic on mental and physical well-being, social connectedness, economic stability, and resilience at provincial, regional, and local levels.
- Results showed that impacts were extensive and widespread, inequitably distributed, with greater impacts for subpopulations experiencing pre-existing disparities.
- The survey methodology provides a framework for developing rapid population health assessments to inform and prioritize public health interventions, practices, and policies.

What are the key implications for public health interventions, practice, or policy?

- The COVID-19 pandemic has exacerbated inequities and existing frailties within healthcare and society, disproportionately affecting some groups, such as young adults, who are not often identified as experiencing health inequities.
- Rapid large-scale population surveys can contribute to and inform prioritization and targeting public health interventions, practices, and policies and demonstrate the need for ongoing surveillance through short- and longer-term recovery.
- Consistent methodology used to develop the surveys, grounded within the social determinants of health, provides a framework for developing future population health assessments.

## Appendix 1. Round one recruitment targets and sample size estimates

**Table:**

Round one: target for BC by HA — rolling up from HSDA by sex by age targets		Responses received	Crude target	% crude target reached	Minimum required target	% minimum target reached	Integrated target	% integrated target reached
Health authority		6012	1753	343.0%	2269	265.0%	2463	244.1%
Interior	Female: 18 to 34 years							
	Female: 35 to 54 years	13,506	2603	518.9%	2313	583.9%	3043	443.8%
	Female: 55 to 74 years	15,710	3264	481.3%	2328	674.8%	3572	439.8%
	Female: 75 years and over	2025	896	226.0%	2146	94.4%	2146	94.4%
	Male: 18 to 34 years	1749	1813	96.5%	2274	76.9%	2484	70.4%
	Male: 35 to 54 years	4187	2474	169.2%	2311	181.2%	2922	143.3%
	Male: 55 to 74 years	6707	3105	216.0%	2326	288.3%	3414	196.5%
	Male: 75 years and over	1444	847	170.5%	2130	67.8%	2130	67.8%
	**HA total**	**51,340**	**16,755**	**306.4%**	**18,097**	**283.7%**	**22,174**	**231.5%**
Fraser	Female: 18 to 34 years	14,779	3901	378.9%	1779	830.7%	3901	378.9%
	Female: 35 to 54 years	29,401	5236	561.5%	1785	1647.1%	5236	561.5%
	Female: 55 to 74 years	24,124	4197	574.8%	1781	1354.5%	4197	574.8%
	Female: 75 years and over	3268	1145	285.4%	1737	188.1%	1737	188.1%
	Male: 18 to 34 years	5471	4005	136.6%	1781	307.2%	4005	136.6%
	Male: 35 to 54 years	11,337	4841	234.2%	1784	635.5%	4841	234.2%
	Male: 55 to 74 years	11,400	3913	291.3%	1781	640.1%	3913	291.3%
	Male: 75 years and over	2200	949	231.8%	1724	127.6%	1724	127.6%
	**HA total**	**101,980**	**28,187**	**361.8%**	**14,152**	**720.6%**	**29,554**	**345.1%**
Vancouver Coastal	Female: 18 to 34 years	15,802	2945	536.6%	1767	894.3%	3075	513.9%
	Female: 35 to 54 years	26,654	3546	751.7%	1778	1499.1%	3546	751.7%
	Female: 55 to 74 years	20,509	2878	712.6%	1773	1156.7%	2942	697.1%
	Female: 75 years and over	3434	874	392.9%	1710	200.8%	1710	200.8%
	Male: 18 to 34 years	7757	2927	265.0%	1768	438.7%	3059	253.6%
	Male: 35 to 54 years	13,362	3239	412.5%	1773	753.6%	3323	402.1%
	Male: 55 to 74 years	10,618	2625	404.5%	1770	599.9%	2750	386.1%
	Male: 75 years and over	2274	696	326.7%	1690	134.6%	1690	134.6%
	**HA total**	**100,410**	**19,730**	**508.9%**	**14,029**	**715.7%**	**22,095**	**454.4%**
Vancouver Island	Female: 18 to 34 years	9331	1682	554.8%	1742	535.6%	2009	464.5%
	Female: 35 to 54 years	20,987	2407	871.9%	1761	1191.8%	2569	816.9%
	Female: 55 to 74 years	27,765	2996	926.7%	1769	1569.5%	3035	914.8%
	Female: 75 years and over	4569	827	552.5%	1690	270.4%	1690	270.4%
	Male: 18 to 34 years	3196	1700	188.0%	1742	183.5%	2012	158.8%
	Male: 35 to 54 years	7261	2223	326.6%	1758	413.0%	2410	301.3%
	Male: 55 to 74 years	12,497	2772	450.8%	1767	707.2%	2825	442.4%
	Male: 75 years and over	3226	746	432.4%	1679	192.1%	1679	192.1%
	**HA total**	**88,832**	**15,353**	**578.6%**	**13,908**	**638.7%**	**18,229**	**487.3%**
Northern	Female: 18 to 34 years	2210	873	253.2%	1692	130.6%	1692	130.6%
	Female: 35 to 54 years	4404	1114	395.3%	1709	257.7%	1709	257.7%
	Female: 55 to 74 years	3455	947	364.8%	1682	205.4%	1682	205.4%
	Female: 75 years and over	315	195	161.5%	1363	23.1%	1363	23.1%
	Male: 18 to 34 years	542	911	59.5%	1696	32.0%	1696	32.0%
	Male: 35 to 54 years	1278	1128	113.3%	1709	74.8%	1709	74.8%
	Male: 55 to 74 years	1330	1032	128.9%	1692	78.6%	1692	78.6%
	Male: 75 years and over	193	194	99.5%	1343	14.4%	1343	14.4%
	**HA total**	**13,727**	**6394**	**214.7%**	**12,886**	**106.5%**	**12,886**	**106.5%**
**BC total**		**356,289**	**86,419**	**412.3%**	**73,072**	**487.6%**	**10,4938**	**339.5%**

**Table:**

Round one: target for BC — rolling up from all HSDA × education targets		Responses received	Crude target	% crude target reached	Minimum required target	% minimum target reached	Integrated target	% integrated target reached
Educational level	Below high school	7090	11,310	62.7%	9311	76.1%	12,811	55.3%
	High school	53,247	26,179	203.4%	9458	563.0%	26,297	202.5%
	Certificate/diploma below bachelor level	120,615	28,042	430.1%	9478	1272.6%	28,072	429.7%
	University degree	173,269	20,887	829.6%	9322	1858.7%	22,365	774.7%
**BC total**		**354,221**	**86,418**	**409.9%**	**37,569**	**942.9%**	**89,545**	**395.6%**

**Table:**

Round one: target for BC — rolling up from all HSDA × income targets		Responses received	Crude target	% crude target reached	Minimum required target	% minimum target reached	Integrated target	% integrated target reached
Income level	< $20,000	10,626	15,243	69.7%	9375	113.3%	16,204	65.6%
	$20,000 to $39,999	29,113	15,918	182.9%	9401	309.7%	16,402	177.5%
	$40,000 to $59,999	41,261	13,066	315.8%	9338	441.9%	14,365	287.2%
	$60,000 to $79,999	43,849	10,382	422.4%	9229	475.1%	12,448	352.3%
	$80,000 to $99,999	40,601	8084	502.2%	9052	448.5%	11,320	358.7%
	$100,000 +	142,780	23,724	601.8%	9161	1558.6%	26,459	539.6%
**BC total**		**308,230**	**86,417**	**356.7%**	**55,556**	**554.8%**	**97,198**	**317.1%**

**Table:**

Round one: target for BC — rolling up from all HSDA × visible minority targets		Responses received	Crude target	% crude target reached	Minimum required target	% minimum target reached	Integrated target	% integrated target reached
Visible minority	Indigenous	9180	5021	182.8%	8864	103.6%	8933	102.8%
	Arab	1170	287	407.7%	3558	32.9%	3558	32.9%
	Black	797	636	125.3%	5658	14.1%	5658	14.1%
	Chinese	15,282	8432	181.2%	6951	219.9%	12,174	125.5%
	Filipino	3135	2306	135.9%	6936	45.2%	6939	45.2%
	Japanese	1497	684	218.9%	5488	27.3%	5488	27.3%
	Korean	1063	973	109.2%	4846	21.9%	4846	21.9%
	Latin American	3027	769	393.6%	5364	56.4%	5364	56.4%
	South Asian	8266	5835	141.7%	7560	109.3%	10073	82.1%
	Southeast Asian	536	883	60.7%	5243	10.2%	5243	10.2%
	West Asian	1658	821	201.9%	3921	42.3%	3921	42.3%
	Multiple visible minorities	9139	496	1842.5%	4179	218.7%	4179	218.7%
	Other	8775	140	6267.9%	2768	317.0%	2768	317.0%
	Not a visible minority	9180	5021	182.8%	8864	103.6%	8933	102.8%
**BC total**		**342,474**	**86,416**	**396.3%**	**80,875**	**423.5%**	**138,277**	**247.7%**

**Table:**

Round one: target for BC — rolling up from HSDA by sex by age targets		Responses received	Crude target	% crude target reached	Minimum required target	% minimum target reached	Integrated target	% integrated target reached
Sex and age	Female: 18–34 years	48,134	11,154	431.5%	9249	520.4%	13,140	366.3%
	Female: 35–54 years	94,952	14,906	637.0%	9346	1016.0%	16,103	589.7%
	Female: 55–74 years	91,563	14,282	641.1%	9333	981.1%	15,428	593.5%
	Female: 75 years and over	13,611	3937	345.7%	8646	157.4%	8646	157.4%
	Male: 18–34 years	18,715	11,356	164.8%	9261	202.1%	13,256	141.2%
	Male: 35–54 years	37,425	13,905	269.1%	9335	400.9%	15,205	246.1%
	Male: 55–74 years	42,552	13,447	316.4%	9336	455.8%	14,594	291.6%
	Male: 75 years and over	9337	3432	272.1%	8566	109.0%	8566	109.0%
**BC total**		**356,289**	**86,419**	**412.3%**	**73,072**	**487.6%**	**104,938**	**339.5%**

## Appendix 2. Round two recruitment targets and sample size estimates

**Table:**

Round two: target for BC by HA — rolling up from HSDA by sex by age targets		Responses received	Crude target	% crude target reached	Minimum required target	% minimum target reached	Integrated target	% integrated target reached
Health authority		1851	1753	105.6%	2269	81.6%	2463	75.2%
Interior	Female: 18 to 34 years							
	Female: 35 to 54 years	5631	2603	216.3%	2313	243.5%	3043	185.0%
	Female: 55 to 74 years	8724	3264	267.3%	2328	374.7%	3572	244.2%
	Female: 75 years and over	1104	896	123.2%	2146	51.4%	2146	51.4%
	Male: 18 to 34 years	513	1813	28.3%	2274	22.6%	2484	20.7%
	Male: 35 to 54 years	1682	2474	68.0%	2311	72.8%	2922	57.6%
	Male: 55 to 74 years	3880	3105	125.0%	2326	166.8%	3414	113.6%
	Male: 75 years and over	814	847	96.1%	2130	38.2%	2130	38.2%
	**HA total**	**24,199**	**16,755**	**144.4%**	**18,097**	**133.7%**	**22,174**	**109.1%**
Fraser	Female: 18 to 34 years	4521	3901	115.9%	1779	254.1%	3901	115.9%
	Female: 35 to 54 years	12,326	5236	235.4%	1785	690.5%	5236	235.4%
	Female: 55 to 74 years	13,038	4197	310.7%	1781	732.1%	4197	310.7%
	Female: 75 years and over	1710	1145	149.3%	1737	98.4%	1737	98.4%
	Male: 18 to 34 years	1692	4005	42.2%	1781	95.0%	4005	42.2%
	Male: 35 to 54 years	4491	4841	92.8%	1784	251.7%	4841	92.8%
	Male: 55 to 74 years	5610	3913	143.4%	1781	315.0%	3913	143.4%
	Male: 75 years and over	1246	949	131.3%	1724	72.3%	1724	72.3%
	**HA total**	**44,634**	**28,187**	**158.3%**	**14,152**	**315.4%**	**29,554**	**151.0%**
Vancouver Coastal	Female: 18 to 34 years	5892	2945	200.1%	1767	333.4%	3075	191.6%
	Female: 35 to 54 years	12,226	3546	344.8%	1778	687.6%	3546	344.8%
	Female: 55 to 74 years	11,331	2878	393.7%	1773	639.1%	2942	385.1%
	Female: 75 years and over	2000	874	228.8%	1710	117.0%	1710	117.0%
	Male: 18 to 34 years	2469	2927	84.4%	1768	139.6%	3059	80.7%
	Male: 35 to 54 years	5479	3239	169.2%	1773	309.0%	3323	164.9%
	Male: 55 to 74 years	5484	2625	208.9%	1770	309.8%	2750	199.4%
	Male: 75 years and over	1323	696	190.1%	1690	78.3%	1690	78.3%
	**HA total**	**46,204**	**19,730**	**234.2%**	**14,029**	**329.3%**	**22,095**	**209.1%**
Vancouver Island	Female: 18 to 34 years	3208	1682	190.7%	1742	184.2%	2009	159.7%
	Female: 35 to 54 years	9357	2407	388.7%	1761	531.3%	2569	364.2%
	Female: 55 to 74 years	15,951	2996	532.4%	1769	901.7%	3035	525.6%
	Female: 75 years and over	2536	827	306.7%	1690	150.1%	1690	150.1%
	Male: 18 to 34 years	1094	1700	64.4%	1742	62.8%	2012	54.4%
	Male: 35 to 54 years	2972	2223	133.7%	1758	169.1%	2410	123.3%
	Male: 55 to 74 years	6718	2772	242.4%	1767	380.2%	2825	237.8%
	Male: 75 years and over	1794	746	240.5%	1679	106.8%	1679	106.8%
	**HA total**	**43,630**	**15,353**	**284.2%**	**13,908**	**313.7%**	**18,229**	**239.3%**
Northern	Female: 18 to 34 years	585	873	67.0%	1692	34.6%	1692	34.6%
	Female: 35 to 54 years	1750	1114	157.1%	1709	102.4%	1709	102.4%
	Female: 55 to 74 years	1676	947	177.0%	1682	99.6%	1682	99.6%
	Female: 75 years and over	167	195	85.6%	1363	12.3%	1363	12.3%
	Male: 18 to 34 years	185	911	20.3%	1696	10.9%	1696	10.9%
	Male: 35 to 54 years	450	1128	39.9%	1709	26.3%	1709	26.3%
	Male: 55 to 74 years	615	1032	59.6%	1692	36.3%	1692	36.3%
	Male: 75 years and over	78	194	40.2%	1343	5.8%	1343	5.8%
	**HA total**	**5506**	**6394**	**86.1%**	**12,886**	**42.7%**	**12,886**	**42.7%**
**BC total**		**164,173**	**86,419**	**190.0%**	**73,072**	**224.7%**	**10,4938**	**156.4%**

**Table:**

Round two: target for BC — rolling up from all HSDA × education targets		Responses received	Crude target	% crude target reached	Minimum required target	% minimum target reached	Integrated target	% integrated target reached
Educational level	Below high school	2250	11310	19.9%	9311	24.2%	12,811	17.6%
	High school	20,266	26,179	77.4%	9458	214.3%	26,297	77.1%
	Certificate/diploma below bachelor level	54,561	28,042	194.6%	9478	575.7%	28,072	194.4%
	University degree	85,501	20,887	409.4%	9322	917.2%	22,365	382.3%
**BC total**		**162,578**	**86,418**	**188.1%**	**37,569**	**432.7%**	**89,545**	**181.6%**

**Table:**

Round two: target for BC — rolling up from all HSDA × income targets		Responses received	Crude target	% crude target reached	Minimum required target	% minimum target reached	Integrated target	% integrated target reached
Income level	< $20,000	3325	15,243	21.8%	9375	35.5%	16,204	20.5%
	$20,000 to $39,999	11,127	15,918	69.9%	9401	118.4%	16,402	67.8%
	$40,000 to $59,999	16,996	13,066	130.1%	9338	182.0%	14,365	118.3%
	$60,000 to $79,999	19,977	10,382	192.4%	9229	216.5%	12,448	160.5%
	$80,000 to $99,999	19,034	8084	235.5%	9052	210.3%	11,320	168.1%
	$100,000 +	72,230	23,724	304.5%	9161	788.5%	26,459	273.0%
**BC total**		**142,689**	**86,417**	**165.1%**	**55,556**	**256.8%**	**97,198**	**146.8%**

**Table:**

Round two: target for BC — rolling up from all HSDA × visible minority targets		Responses received	Crude target	% crude target reached	Minimum required target	% minimum target reached	Integrated target	% integrated target reached
Visible minority	Indigenous	4798	5021	95.6%	8864	54.1%	8933	53.7%
	Arab	136,264	59,133	230.4%	9539	1428.5%	59,133	230.4%
	Black	6089	8432	72.2%	6951	87.6%	12,174	50.0%
	Chinese	2873	5835	49.2%	7560	38.0%	10,073	28.5%
	Filipino	537	636	84.4%	5658	9.5%	5658	9.5%
	Japanese	1244	2306	53.9%	6936	17.9%	6939	17.9%
	Korean	1228	769	159.7%	5364	22.9%	5364	22.9%
	Latin American	461	883	52.2%	5243	8.8%	5243	8.8%
	South Asian	193	287	67.2%	3558	5.4%	3558	5.4%
	Southeast Asian	622	821	75.8%	3921	15.9%	3921	15.9%
	West Asian	362	973	37.2%	4846	7.5%	4846	7.5%
	Multiple visible minorities	1011	684	147.8%	5488	18.4%	5488	18.4%
	Other	1180	496	237.9%	4179	28.2%	4179	28.2%
	Not a visible minority	4668	140	3334.3%	2768	168.6%	2768	168.6%
**BC total**		**161,530**	**86,416**	**186.9%**	**80,875**	**199.7%**	**138,277**	**116.8%**

**Table:**

Round two: target for BC — rolling up from HSDA by sex by age targets		Responses received	Crude target	% crude target reached	Minimum required target	% minimum target reached	Integrated target	% integrated target reached
Sex and age	Female: 18–34 years	16,057	11,154	144.0%	9249	173.6%	13,140	122.2%
	Female: 35–54 years	41,290	14,906	277.0%	9346	441.8%	16,103	256.4%
	Female: 55–74 years	50,720	14,282	355.1%	9333	543.4%	15,428	328.8%
	Female: 75 years and over	7517	3937	190.9%	8646	86.9%	8646	86.9%
	Male: 18–34 years	5953	11,356	52.4%	9261	64.3%	13,256	44.9%
	Male: 35–54 years	15,074	13,905	108.4%	9335	161.5%	15,205	99.1%
	Male: 55–74 years	22,307	13,447	165.9%	9336	238.9%	14,594	152.9%
	Male: 75 years and over	5255	3432	153.1%	8566	61.3%	8566	61.3%
**BC total**		**164,173**	**86,419**	**190.0%**	**73,072**	**224.7%**	**104,938**	**156.4%**

## Appendix 3. Statistical weighting calculations

Geography (HSDA, LHA, CHSA)-specific survey weights for analysis using the 2016 Canadian Census data for reference were developed. The weights were defined as the census proportion divided by the observed sample proportion for each corresponding demographic combination. The final geography-specific survey weights were derived based on the data available in the following hierarchy:

- Age group, sex, education and visible minority
- Age group, sex, education
- Age group, sex, visible minority
- Age group, sex

where the values of each demographic are as follows:

- Age group: 18 to 34 years, 35 to 54 years, 55 to 74 years, 75 years and over
- Sex: male or female
- Education: below high school, high school, certificate or diploma below bachelor level, university degree
- Visible minority: white, Indigenous, Chinese, South Asian, others

In round one, LHA-specific survey weights were applied in all analyses at BC, HA, HSDA, and LHA as less than 40 respondents with missing LHA survey weights. The CHSA-specific survey weights were used for analyses at the CHSA level.

In round two, HSDA-specific survey weights were applied for analyses at BC, HA, or HSDA level, whereas LHA- or CHSA-specific survey weights were used for analyses at LHA or CHSA level, respectively.
